# Emerging Disparities in Prevention and Survival Outcomes for Patients with Head and Neck Cancer and Recommendations for Health Equity

**DOI:** 10.1007/s11912-022-01273-5

**Published:** 2022-04-14

**Authors:** Manisha Salinas, Ashish Chintakuntlawar, Ivie Arasomwan, Ahmed Eltahir, Katharine A. R. Price

**Affiliations:** 1 Department of Hematology and Oncology, Mayo Clinic, 4500, San Pablo Rd S., Jacksonville, FL 32224 USA; 2 Department of Hematology and Oncology, Mayo Clinic, 200 First Street SW, Rochester, MN 55905 USA; 3 Department of Otolaryngology, Head and Neck Surgery, Mayo Clinic, Rochester, MN USA

**Keywords:** Cancer disparities, Head and neck cancer, Health equity, Rurality, Nutrition, Telehealth, Digital health, HPV vaccination

## Abstract

**Purpose of Review:**

The aim of this review is to describe less known and emerging disparities found in the prevention and survival outcomes for patients with head and neck cancer (HNC) that are likely to play an increasingly important role in HNC outcomes and health inequities.

**Recent Findings:**

The following factors contribute to HNC incidence and outcomes: (1) the effect of rurality on prevention and treatment of HNC, (2) dietary behavior and nutritional factors influencing the development of and survival from HNC, and (3) barriers and benefits of telehealth for patients with HNC.

**Summary:**

Rurality, nutrition and diet, and telehealth usage and access are significant contributors to the existing health disparities associated with HNC. Population and culturally specific interventions are urgently needed as well as more research to further define the issues and develop appropriate population and individual level solutions.

## Introduction

It is estimated that in 2021 more than 68,000 individuals in the United States were diagnosed with head and neck cancer (HNC) including cancers of the oral cavity, oropharynx, hypopharynx, and larynx [1]. HNC is the seventh most common cancer globally and accounts for 3% of cancer deaths worldwide, with a 40–50% 5-year-survival rate [1]. While there have been advances in the prevention, treatment, and survival of patients with HNC, significant disparities continue to exist across the cancer continuum.

Known major risk factors for HNC include smoking, alcohol use, and chronic human papillomavirus (HPV) infection. Socioeconomic status, education, age, and income significantly impact risk for developing HNC [2]. Health disparities among vulnerable populations amplify risk factors and increase the burden on specific groups with limited resources. For example, studies examining racial disparities in HNC found Black patients as being less likely to receive medical care, despite having signs of HNC. Marginalized groups bear an unequal burden of disease and risk for more adverse outcomes due to factors such as likelihood of comorbidities, historical discrimination in medical systems, delays in diagnoses and subsequent treatment for advanced stages, and lack of support and shared decision-making approaches when navigating complex medical situations [3, 4, 5]. In addition to this myriad of factors which are well-established in their contribution to HNC, it is important to examine other existing disparities which may be alleviated through further investigation and appropriate tailored interventions.

Although the incidence of HNC overall has declined in the USA, with significant declines noted in non-oropharyngeal cancers in black men and women [6], significant pockets of disparities in HNC incidence and outcomes persist [5]. The aim of this review is to describe less known and emerging disparities found in prevention and survival outcomes for patients with HNC that are likely to play an increasingly important role in cancer outcomes and cancer-related health inequity. Prevalent determinants uncovered included (1) the effect of rurality on prevention and treatment of HNC, (2) dietary behavior and nutritional factors influencing the development of and survival from HNC, and (3) barriers and benefits of telehealth for patients with HNC. We chose to focus on these three emerging disparities as they are less well-described yet augment and intersect with the existing known health disparities pertinent to patients with HNC. Additionally, recommendations for a holistic and equitable approach to HNC prevention and treatment are proposed with a focus on the described emerging disparities.

## Rurality

Health disparities between urban and rural populations are increasingly being recognized and addressing cancer-associated disparities in rural populations is a priority for the National Cancer Institute. Rurality provides context for understanding behavioral and environmental health inequities, reaffirms that “place matters”, and is a major driver of HNC disparities across the disease spectrum from primary prevention to survival outcomes. [7]

### Incidence and Prevention

The trends in incidence of HNC are found to be unfavorable in rural areas compared with urban areas, and lifestyle risk factors for the development of HNC put rural populations at disproportionate risk with higher rates of tobacco and alcohol use and lower rates of HPV vaccination. A study using the Surveillance, Epidemiology, and End Results (SEER) database analyzed incidence rates of cancer from 1973 through 2015 and found that the incidence of larynx and oral cavity cancer—both tobacco and alcohol driven—are decreasing faster in urban areas than rural, with oral cavity cancer incidence changing over the decades from substantially lower in rural areas to substantially higher compared with urban areas [8]. Data from the Center for Disease Control and Prevention demonstrates that non-metropolitan rural counties in the USA have a higher incidence and death rate from smoking-related cancers including larynx cancer. [9••]

HPV now causes most new cases of HNC in the USA and represents a distinctly different disease than smoking- and alcohol-associated HNC with different risk factors, a more favorable biology, and a higher cure rate [10]. HPV-HNC is typically diagnosed in patients at a younger age, thus impacting patients who are active participants in the workforce with resultant economic morbidity on individuals and communities [11]. Multiple studies have shown that the rate of development of HPV-associated HNC is higher in rural than urban settings [12, 13]. Incidence of HPV-associated oropharynx (tonsil and base of tongue) cancer continues to rise and is now the eighth most common cancer in men in the USA; however, the incidence rates have started to level off in urban areas but continue to sharply rise in rural areas [1, 8]. HPV-driven cancers represent a significant opportunity for cancer prevention given the availability of highly effective vaccination against HPV, yet disparities in acceptance and completion of the HPV vaccine series have confounded HPV vaccine utilization [14]. Although the HPV vaccine has been available in the USA for 15 years, vaccination rates overall still remain below the target of 80%, and are significantly lower in rural compared with metropolitan areas [15, 16]. Racial inequities in vaccine administration continue to persist; a systematic review and meta-analysis found that compared to whites, racial minorities have higher rates of vaccine initiation but less rates of completion of the full HPV vaccine series [17]. The gender disparity in HPV vaccination rates among girls compared with boys has been longstanding since the vaccination was initially introduced in 2007 for girls only. Despite consensus recommendations since 2009 that both girls and boys receive the HPV vaccination series, boys have been consistently vaccinated at a lower rate than girls across the USA. The gender gap in HPV vaccination is magnified in the rural setting, with the HPV vaccination rates of boys ages 13–17 only 5.31% and 5.5% in isolated small rural towns and rural towns, respectively, compared with 30% and 26.8% in girls [15, 18]. A recent study demonstrated that rates of HPV vaccination could be improved in a rural setting with interventions including healthcare team training activities and distribution of patient education materials along with technology-based patient HPV vaccination reminders for parents and caregivers and young adult patients [19]. The combination of low vaccination rates and rising HPV oropharynx cancer cases in rural areas sets the stage for a worsening health disparity for decades unless urgent attention is paid to increasing HPV vaccination in rural communities.

Growing rates of obesity and low physical activity in rural areas can increase the risk for cancer and contribute to health inequities [20]. Furthermore, the limited consumption of healthy foods and growing food insecurity are concerning as risk factors for cancer and poor health outcomes in rural settings [21]. Although a clear association between obesity and overall HNC incidence and survival has not been clearly demonstrated,^22,23^ there are subsets of patients with HNC who are at risk. Notably, obese patients (BMI ≥30 kg/m^2^) with early-stage tongue cancer who underwent curative-intent treatment had inferior disease-specific survival and recurrence-free survival than non-obese patients [24]. Physical activity is being increasingly recognized as playing a role in carcinogenesis of HNC [25, 26]. Dietary patterns of rural versus non-rural populations are varied but decreased consumption of fruits and vegetables in rural areas has been described and could contribute to the increased risk of HNC in patients living in non-urban settings (see “Nutrition” section) [27]. Food insecurity represents a substantial risk factor that is magnified in rural settings, and places individuals at risk for cancer. [21, 28] Food insecurity has been associated with poor nutrition and diets that are lower in fruits and vegetables. [28] The intersection of food insecurity, lack of healthy food, and physical inactivity, like many existing health disparities, impact minority populations and communities of color disproportionately, and this effect is further magnified in rural communities. Collectively, rurality drives these issues and puts rural populations at higher risk for the development of HNC. [29, 30]

### Survival Outcomes

Significant disparities in survival of patients with HNC are seen when comparing rural to urban populations, and rurality magnifies co-existing racial disparities in HNC survival [31, 32]. A large retrospective study of over 146,000 patients with HNC through the National Cancer Database showed significant survival differences among white patients who lived in urban areas (median survival 67 months) compared with white patients in rural areas (median survival 59.1 months) [32]. Similar results were seen for the Black patient population with a median survival for Black urban patients 43.1 months and 35.1 months for Black rural patients. The intersection of race and rurality is highlighted in this study as the survival of white rural patients was still higher than that of Black urban or rural patients. For individuals who are HNC survivors, those living in rural areas are more likely to have lower health-related quality of life than urban patients, and suicide rates for rural patients with HNC are higher than the general population and those in urban areas. [33•, 34]

The impact of rurality on survival outcomes for patients with HNC is multifactorial. One contributing factor is that patients with HNC living in a rural setting are often diagnosed at a more advanced stage, resulting in poorer survival rates. A recent study demonstrated the link of low socioeconomic status and higher T- and N-stage tumors in patients with HNC who were categorized as rural residents [35]. A retrospective study of patients treated for HNC in Southeastern USA showed that patients living in rural areas presented more often with stage III–IV cancer compared with their urban counterparts [36]. Another contributing factor to the rural-urban gap in survival for patients with HNC is less access to healthcare, and specifically less access to tertiary referral centers or academic centers where outcomes for HNC treatment have clearly been shown to be superior to those of small community hospitals [37, 38, 39]. In a recent study, it was found that most patients treated in academic centers had greater survival, and black rural residents had lower survival outcomes [40]. Recent data presented at the Annual Society of Clinical Oncology Meeting in 2021, however, suggests that if patients with HNC in rural and urban settings are treated with similar treatment protocols that survival outcomes can be the same, supporting the idea that efforts to improve timely access to care at appropriate medical facilities is critical. [41]

Increased travel time to treatment hospitals puts additional burden on those living in rural, less accessible areas [42]. While it has been found that traveling longer distance for HNC treatment results in better outcomes, racial minorities, such as Black and Hispanic groups, are less likely to travel longer distances, and therefore less likely to receive treatment from academic and high-volume centers. [43•] Notably, rural-urban differences in survival outcomes for HNC were not seen in a study of patients treated through a single-payer, government-funded universal healthcare system suggesting a more comprehensive, universal healthcare insurance program would be of significant benefit to rural residents diagnosed with HNC. [44]

## Nutrition

A whole-food, plant-based diet is one of the most important tools to prevent cancer yet remains an under-utilized strategy to improve cancer-related outcomes. Plant-based diets impact chronic disease including cancer by decreasing chronic inflammation and harnessing the power of phytochemicals—biologically active substances in plants—that can impact all the major signaling pathways leading to carcinogenesis including but not limited to inflammation, immunity, apoptosis, cell replication, and angiogenesis [45]. Interventions to improve the diet of all patients, particularly those at risk for malnutrition and food insecurity such as minority populations and those living in rural areas, could help alleviate health disparities including those related to HNC.

### Incidence and Primary Prevention

Numerous population-based cohort studies from around the world have shown that diets high in vegetables and fruits significantly decrease the risk of developing head and neck squamous cell carcinoma [46, 47, 48, 49, 50, 51, 52]. In addition, it has been shown that consumption of a diversity of vegetables and fruits can decrease the risk of developing cancers of the larynx, oral cavity, and pharynx by up to 60% [53]. A meta-analysis of epidemiologic studies from 2002 concluded “There is enough evidence to point to a preventive role of vegetable intake, including green vegetables, cruciferous vegetables, and yellow vegetables, total fruit intake, and citrus fruit intake,” yet dietary factors remain an overlooked and under-discussed risk factor for HNC [50]. Promoting nutrient-rich diets and recommending incremental and manageable modifications can be especially useful for survivors to prevent HNC recurrence and improve healthy day-to-day living. [54]

HPV-associated oropharynx cancer has an improved prognosis with high cure rates compared with non-HPV HNC [10]. The improved outcomes for HPV-HNC have been attributed to factors including fewer tumor mutations, increased chemotherapy and radiation-sensitivity, and patients with fewer medical comorbidities. However, it has been proposed that the improved nutritional status of patients with HPV oropharynx cancer could play a role in the better survival outcomes compared with HPV-negative tumors. Researchers at the University of Michigan investigated the association of HPV status and twelve micronutrients in a cohort of patients treated for head and neck squamous cell carcinoma. They found that several micronutrients (vitamin A, vitamin E, iron, β-carotene, and folate) were associated with HPV-positive status even after controlling for confounding factors including age, sex, tumor site and stage, and alcohol and tobacco use, suggesting that diet may be a factor in the improved prognosis of patients with HPV-HNC. [55]

### Secondary Prevention of HNC Recurrence

A growing body of evidence supports the idea that a diet enriched in vegetables and fruits may decrease the risk of recurrence of HNC after curative-intent treatment. A hospital-based prospective study in Spain, using pre-and post-treatment food frequency questionnaires, showed that high vegetable consumption pre-treatment was significantly associated with reduced cancer recurrence and overall mortality, and high vegetable consumption post-treatment was significantly associated with reduced cancer recurrence, overall mortality, and oral cancer mortality [56]. A multi-center US case-control study of patients with oral cavity or oropharynx cancer also showed a trend towards decreased incidence of second primary cancers of the head and neck in patients with high total vegetable intake, and increased consumption of dark yellow vegetables, cruciferous vegetables, and leafy greens [57]. A prospective study of 934 patients with newly diagnosed HNC showed a trend towards decreased mortality in patients who followed a whole-food diet with high intake of vegetables, fruit, legumes, fish, poultry, whole grains, fruit juice, olive oil, nuts, and garlic compared with patients who consumed a western diet characterized by high intake of red and processed meats, refined grains, French fries, potatoes, condiments, high-fat dairy products, eggs, coffee, desserts, snacks, mayonnaise [58]. A diet high in fruits and vegetables is inherently high in fiber, which could help prolong survival in patients with HNC. [59]

### Vitamin D and Risk of Head and Neck Cancer

Vitamin D is an emerging dietary risk factor that could play a role in the development of and survival from HNC. It has been previously recognized that patients with HNC with lower levels of vitamin D were at increased risk for cancer recurrence [60]. A large meta-analysis of 16 studies and over 81,000 participants reported a 32% decrease in HNC incidence in patients with high concentrations of circulating 25-hydroxyvitamin D and an increase in survival for participants with HNC with higher concentrations of vitamin D (hazard ratio 1.13, 1.05–1.22) [61]. Racial disparities in vitamin D levels have been documented with blacks frequently experiencing hypovitaminosis D compared with whites [62, 63]. A recent small study of patients with HNC reported lower circulating levels of vitamin D in black versus white patients [64]. Rural residence has also been shown to be associated with lower levels of vitamin D [65]. The primary sources of vitamin D are sun exposure, fortified foods, and supplementation; clear, culturally tailored messaging to increase outdoor activity and access to food sources of vitamin D could potentially be a tool to prevent HNC. However, a causal relationship between vitamin D and HNC has not been established, and until there is evidence from a prospective, randomized clinical trial, supplementation of vitamin D should not be routinely recommended for the purpose of HNC prevention. Rather, it could be a component of a whole-food diet and part of recommendations that support healthy lifestyle choices.

### *Nutritional Disparities in Patients with HNC*

While improvement in overall nutritional health has proven to be an important achievement of public health efforts to promote healthy dietary patterns and prevent chronic diseases and cancers, disparities continue to persist among various populations, including rural or geographically isolated, and ethnic minorities [66, 67]. A recent study found that patients with a history of throat cancer are more likely to live in areas with food insecurity, especially among Black and Hispanic groups [68]. Increasing screenings for food insecurity among patients with HNC, especially among patients from disadvantaged groups, is needed to improve equal access to care, which can be further exacerbated by socioeconomic status, income, race and ethnicity, and geographical residence [66]. Poverty, often associated with food insecurity and malnutrition, can also impact survival outcomes among HNC patients, particularly for persons of color, in rural residences, and those who are of older ages [69]. Findlay and colleagues found that implementing a dietic model with a HNC multidisciplinary team resulted in positive health outcomes, saving medical costs and unplanned hospital admissions, and can be one strategy to help address the growing gap from evidence-based guidelines to patient-centered practice with dietary behaviors in HNC [70]. At the onset of the COVID-19 pandemic, growing financial strains have contributed to the widening gap in disparities with food insecurity among the cancer community,^71^ and more current research is needed on the impact of patients with HNC that have been historically disadvantaged, including communities of color (including stratified categories by subgroups), those with low socioeconomic status, and other relevant factors.

## Telehealth

The COVID-19 pandemic has catapulted telehealth to the forefront of modern cancer care with the promise of significant increases in access to care and the potential pitfall of widening existing health disparities. The opportunity to provide virtual visits could mitigate several disparities such as traveling to academic medical centers, wait times in hospitals for consultation, and access for lower socioeconomic groups [72]. For patients with HNC who live in rural areas, strategic use of telehealth services could be a valuable tool to narrow the disparity gap in access to care. Conversely, telehealth care may present barriers for communities which vary on sociodemographic characteristics such as age, race and ethnicity, and residence. For example, among cancer survivors, Jewett et al. found that during the onset and progression of the pandemic, there was less video visit usage among older adults and survivors that were persons of color [73]. The importance of digital health equity requires thoughtful and urgent attention to account for individuals with limited internet connectivity, familial support and safety at home, and updated, reliable devices. [74]

Several studies looking at the use of telehealth in patients with HNC have been published. As early as 2009, a telehealth intervention was designed using feedback from patients and clinicians to counteract patient isolation, develop patient self-efficacy, and improve symptom management [75]. A recent study of patients with HNC during the pandemic found that most patients with HNC preferred in-person visits over virtual ones [76]. Another study found a behavioral physical intervention to manage fatigue among HNC patients has a high potential to be adapted to fit the needs of rural patients through home health or telehealth care [77]. Additionally, Tam et al. found patients with HNC who had no or public insurance types, low household incomes, and lower education level were less likely to engage in virtual visits during the pandemic, but that there is greater likelihood for alleviating some of the disparities through telephone visits [78]. Home-based telehealth care has been found to be effective in previous studies with HNC patients, which improved access and satisfaction with telehealth care, especially among older individuals [79]. With the growing possibilities in digitalized healthcare, patients with HNC may have access to services that could impact early detection of cancer and improve quality of life and engagement in the healthcare system,^80^ but we must remain cognizant of the availability of resources and specific needs and barriers of various groups [81]. Previous literature has shown telehealth in general may be significantly underutilized among patients with limited English proficiency or those who are older, Black or Hispanic, and using Medicaid or Medicare; these factors also play a significant role for patients with HNC. [43•, 82, 83, 84] Early studies showed promise of telehealth improving HNC patient quality of life, [85] and as this population increases in vulnerability, it is important to continue surveillance and communication with survivors and in post-operative care to recognize disparities in telehealth usage [86]. It is necessary to develop tailored strategies to increase access for HNC patients such as maintaining alternatives to telehealth video visits, accounting for available and accessible resources in virtual communication, and giving individualized options for care, during the pandemic and beyond. [81]

## Recommendations for Future Work

In this paper, we have focused on rurality, nutrition, and telehealth as important components of HNC prevention and treatment that can intersect and magnify existing disparities in HNC. Although efforts to reduce disparities in patients with HNC are ongoing, more is needed to address the emerging inequities described here. By focusing on factors such as rural characteristics, dietary patterns, and telehealth use, we call for gaining a deeper understanding of the holistic and societal contributors that impact patients with HNC from prevention through treatment and offer several recommendations to take into consideration for future research and practice.

First and foremost, it is imperative to partner with underrepresented communities to assess their needs and understanding, develop intervention tools, and implement sustainable practices. Community input is critical throughout the process to ensure culturally sensitive and population-specific interventions, and assessment of any intervention through pilot programs, quality-improvement projects, or formal research is needed to measure the impact and success. For all populations, clear messaging to promote HPV vaccination, physical activity, and a whole-food, plant-based diet rich in a diversity of fruits and vegetables are needed and should be the foundation for efforts to prevent HNC. Although not addressed specifically in this paper, tobacco and alcohol cessation remains critical. Diet modification—even minor changes—can improve healthy behaviors and reduce the risk for several cancers [87]. Li et al. found in their population-based study that a consistent, healthy diet based on public health guidelines may lower risk of HNC [88]. Among survivors, it is also important to monitor nutritional intake and dietary patterns, as cancer survivors can be at risk for low physical activity, low nutrient diet, and other behaviors which can negatively impact overall health [89]. In a systematic review, Crowder et al. advocate for long-term follow-up in HNC survivors, as they have demonstrated risk for malnutrition, specifically post-chemoradiotherapy [90]. Research also suggests increasing interventions to specifically address obesity and encourage consumption of fruit and vegetables containing vitamins and carotenoids to reduce HNC risk [91]. Finally, engaging in nutritional counseling to help with nutritional status and clinical outcomes has potential to be beneficial in HNC patients [92], and further including culturally sensitive material and trained counselors to tailor education and messaging towards underrepresented groups could enhance positive outcomes and reduce disparities in care.

Hesitancy surrounding HPV vaccination is multi-factorial, and research to understand the barriers for individual communities is critical [93••]. Data-supported efforts to improve HPV vaccination uptake include same day vaccination, consistency of the recommendation for all individuals, providing a strong and clear recommendation for the vaccine, and emphasizing the cancer prevention benefits [93••]. Further research is needed into the barriers and facilitators of HPV vaccination uptake in rural communities, with timely implementation of the findings to counteract the growing epidemic of HPV-associated HNC in rural communities.

Telehealth is a rapidly changing landscape that needs to be considered and incorporated into all aspects of care and research for cancer patients. Deliberate assessment of diverse communities uses of and access to telehealth is imperative, and further understanding of the HNC population through research including those in rural areas and across racial groups is needed to best learn how to employ telehealth to meet the needs of racial, ethnic, and geographically diverse patients with HNC.

Our recommendations for patients living in rural areas are summarized in Table 1. Health behavior education and implementing ongoing interventions which educate patients on specific dietary habits (whole-food, plant-based diets) as well as exercise and physical activity are foundational. We also call for more efforts to improve HPV vaccine rates through comprehensive health promotional programs. Providing accessible telehealth based on patients’ needs and resources, as well as strategies for assisting with transportations needs, will be crucial to maintain consistency of visits and monitoring during and after treatment. Additionally, larger scale programs which address food insecurity to improve availability of healthy food can help combat various factors which impact outcomes for HNC patients. Lastly, improving the scope of healthcare coverage through universal programs and expanding accessibility to medical centers for specialty care can improve the health outcomes and well-being of patients with HNC.

**Table 1:** Recommendations for strategies to improve prevention and outcomes of head and neck cancer among rural patients

Recommendations for rural populations	
Comprehensive health behavior education	Ongoing interventions aimed to improve education on health behaviors including whole-food, plant-based diet, and physical activity
HPV vaccine promotion	Increase efforts to address HPV vaccine access through comprehensive programs to promote HPV vaccination
Accessible telehealth services	Improve access and long-term reliability of telehealth care, including video visits, for earlier diagnosis, access to care, and follow-up
Reliable transportation	Provide assistance with convenient and reliable transportation to referral centers with expertise in HNC
Food and dietary interventions	Develop and integrate programs in the community to combat food insecurity
Comprehensive healthcare availability	Work towards implementation of state universal health care to improve access and increase the utilization of medical centers with comprehensive services for patients with HNC

## Conclusion

HNC represents a significant health burden with ongoing health disparities. Focusing on rural populations, nutrition, and telehealth efforts could improve HNC outcomes across the cancer continuum. It is important to take these factors into consideration in the changing climate of various structures which contribute to barriers and disparities among diverse groups.
